# A retrospective study of small-pelvis radiotherapy plus image-guided brachytherapy in stage I–II non-bulky cervical squamous cell carcinoma

**DOI:** 10.1093/jrr/rrac001

**Published:** 2022-02-12

**Authors:** Yuya Yoshimoto, Kazutoshi Murata, Daisuke Irie, Ken Ando, Akiko Adachi, Hiroshi Aoki, Takashi Hirakawa, Shin-ei Noda, Takashi Nakano, Tatsuya Ohno

**Affiliations:** Department of Radiation Oncology, Gunma University Graduate School of Medicine, 3-39-22 Showa-machi, Maebashi, Gunma 371-8511, Japan; Department of Radiation Oncology, Fukushima Medical University, 1, Hikarigaoka, Fukushima, Fukushima 960-1247, Japan; Department of Radiation Oncology, Gunma University Graduate School of Medicine, 3-39-22 Showa-machi, Maebashi, Gunma 371-8511, Japan; QST Hospital, National Institute for Quantum Science and Technology (QST), 4-9-1 Anagawa, Inage-ku, Chiba 263-8555, Japan; Department of Radiation Oncology, Gunma University Graduate School of Medicine, 3-39-22 Showa-machi, Maebashi, Gunma 371-8511, Japan; Department of Radiation Oncology, Gunma University Graduate School of Medicine, 3-39-22 Showa-machi, Maebashi, Gunma 371-8511, Japan; Department of Radiation Oncology, Gunma University Graduate School of Medicine, 3-39-22 Showa-machi, Maebashi, Gunma 371-8511, Japan; Department of Obstetrics and Gynecology, National Hospital Organization Takasaki General Medical Center, 36 Takamatsu-cho. Takasaki, Gunma 370-0829, Japan; Department of Obstetrics and Gynecology, Gunma University Graduate School of Medicine, 3-39-22 Showa-machi, Maebashi, Gunma 371-8511, Japan; Department of Radiation Oncology, Saitama Medical University International Medical Center, 1397-1 Yamane, Hidaka, Saitama 350-1298, Japan; Quantum Life and Medical Science Directorate, National Institute for Quantum Science and Technology (QST), 4-9-1 Anagawa, Inage-ku, Chiba 263-8555, Japan; Department of Radiation Oncology, Gunma University Graduate School of Medicine, 3-39-22 Showa-machi, Maebashi, Gunma 371-8511, Japan

**Keywords:** early-stage cervical cancer, definitive radiotherapy, small-pelvis radiotherapy, common iliac lymph node

## Abstract

We herein report a retrospective analysis of the efficacy of a combination therapy of pelvic irradiation that excluded the common iliac lymph nodes region and image-guided brachytherapy (IGBT) for non-bulky (≤4 cm) cervical cancer. Thirty-three patients with stage I–II cervical squamous cell carcinoma (≤4 cm) and without pelvic/para-aortic lymphadenopathy who were treated with definitive radiotherapy alone between February 2009 and September 2016 were included. The radiotherapy consisted of CT-based small-pelvis irradiation (whole pelvis minus common iliac lymph node area) of 20 Gy/10 fractions followed by pelvic irradiation with a midline block of 30 Gy/15 fractions and IGBT of 24 Gy/4 fractions (6 Gy/fraction for high-risk [HR] clinical target volume [CTV] D90%). In-room computed tomography (CT) imaging with applicator insertion was used for brachytherapy planning, with physical examinations and diagnostic magnetic resonance imaging (MRI) also being referred to for determination of HR CTV. Over a median follow-up of 60.5 months (range, 7–89), two patients developed distant recurrence and one developed local and distant recurrence. Two patients died from cervical cancer, one from hepatocellular carcinoma and one from non-cancerous disease. The 2/5-year local control (LC), progression-free survival (PFS) and overall survival (OS) rates were 100%/96.7%, 93.8%/90.6% and 93.9%/93.9%, respectively. No pelvic/para-aortic lymph node recurrence was observed. There were no late complications of grade 3 or higher in the small bowel, large bowel/rectum, or bladder. Our results suggest that a combination therapy of IGBT plus small-pelvis irradiation excluding common iliac lymph nodes provides reasonable clinical outcomes and can be a treatment option in non-bulky (≤4 cm) cervical squamous cell carcinoma.

## INTRODUCTION

Radiotherapy plays an important role in the treatment of cervical cancer and the treatment outcomes for early-stage cervical cancer are extremely good; therefore, efforts should be made to reduce long-term complications without undermining the efficacy of treatment. Radiotherapy for cervical cancer generally consists of a combination of external irradiation and brachytherapy. For brachytherapy, case-by-case optimization procedures using CT-planning and needle insertion have been developed [1, 2].

Traditionally, L4/5 has been widely used for the upper border of the external irradiation field for the pelvis in 2D radiotherapy. An anatomical study showed that the mean level of the aortic bifurcation was 6.7 cm above the lumbosacral prominence [3]. Thus, the border of L4/5 does not sufficiently cover the common iliac lymph node area. Currently, CT-based 3D treatment planning is commonly performed, which requires a larger clinical target volume (CTV) than that of 2D radiotherapy to cover the common iliac lymph node area. Although the JASTRO 2020 guidelines recommended that the common iliac region be included in the radiation field, the risk-based individualized target volumes for external irradiation have not been established.

It was previously reported that a small-pelvis irradiation plan excluding the common iliac lymph node region from conventional radiation fields can be potentially useful for reducing the small bowel dose [4]. A pathological mapping study of surgically-treated patients revealed that the rate of common iliac lymph node metastasis in stage I–II squamous cell carcinoma of the cervix was only 2.4% in cases with a tumor size ≤4 cm, supporting the use of such a small pelvic field plan strategy [5] (Supplementary Table 1). We herein report the results of a combination therapy of pelvic irradiation with exclusion of the common iliac lymph node (the small pelvis) and image-guided brachytherapy (IGBT) for non-bulky (≤ 4 cm) cervical squamous cell carcinoma.

## MATERIALS AND METHODS

### Patient characteristics

A retrospective chart review of 33 consecutive patients with stage I–II non-bulky cervical squamous cell cancer who underwent definitive radiotherapy was performed. The inclusion criteria were as follows: histologically proven cervical squamous cell cancer (≤ 4 cm), no pelvic/para-aortic lymphadenopathy, and treatment with definitive radiotherapy alone between February 2009 and September 2016. Generally, lymph nodes with a short axis diameter of more than 1 cm are defined as positive. FDG-PET studies were also referred to when available (30/33, 90.9%).

### External beam radiotherapy and brachytherapy

**Fig. 1.**
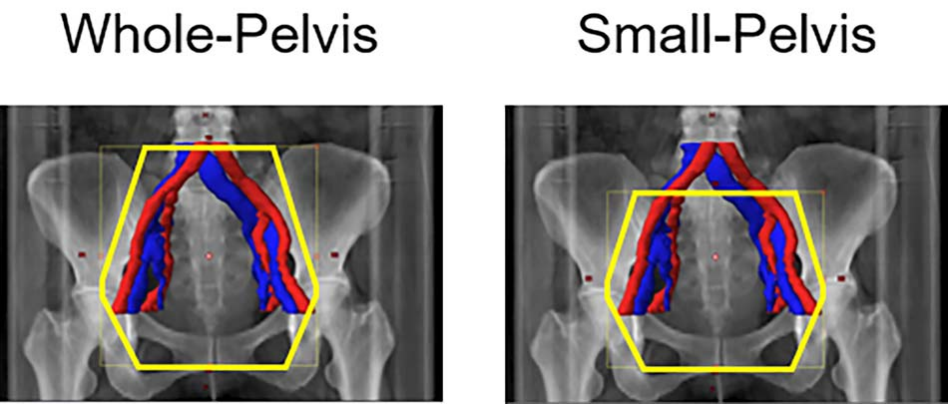
A representative small-pelvis irradiation field. A small-pelvis irradiation plan excludes the common iliac lymph node region from the conventional whole pelvis radiation fields (Ref. 4).

Patients were treated with combined external beam radiotherapy (EBRT) and high-dose-rate brachytherapy [1]. External pelvic irradiation was performed using an anteroposterior/posteroanterior field or the box technique, with doses of 2 Gy per fraction, delivered five times per week. The CTV included the cervical tumor, uterus, parametrium, upper half of the vagina and pelvic lymph node regions without the common iliac lymph node (a small-pelvis field, Fig. 1). Central shielding (CS; 3 cm in width) was inserted at a total dose of 20 Gy. Pelvic irradiation with CS was performed with an anteroposterior/posteroanterior field to a total dose of 50 Gy. Concurrent/adjuvant chemotherapy was not delivered to the patients.

Along with the pelvic CS irradiation, high-dose-rate brachytherapy was performed using an ^192^Ir remote afterloading system (microSelectron, Elekta, Stockholm, Sweden). Four fractions of brachytherapy were administered once a week, with a fraction dose of 6 Gy. One patient with cone biopsy-diagnosed stage IB1 was treated with three fractions of brachytherapy. Magnetic resonance imaging (MRI) was acquired from each patient at diagnosis and within 1 week before the first brachytherapy session. A set of Fletcher-Suit Asian Pacific applicators (tandem and half-size ovoid) were inserted under ultrasound guidance. For patients with asymmetric residual tumors (six of 33 cases, 13 of 131 brachytherapy sessions), trocar point needles (Nucletron; Elekta, Stockholm, Sweden) were additionally inserted in combination with the applicators [6]. After implantation, computed tomography (CT) images were obtained on the same couch and the data were transferred to an Oncentra treatment planning system (Elekta, Stockholm, Sweden) for contouring and planning. The high-risk (HR) CTV and organs at risk (OARs) were contoured according to the recommendations of the Groupe Européen de Curiethérapie and the European Society for Radiotherapy and Oncology [7, 8]. We predicted that a 6 Gy isodose line should cover the HR CTV to achieve an HR CTV D90% (the minimum dose delivered to 90% of the HR CTV) of >6 Gy [9]. HR-CTV D90% >6 Gy was achieved in 117 of 131 brachytherapy sessions (89.3%). The median HR-CTV D90% per patient was 6.89 Gy. With the aim of quantifying changes in dose volume histogram (DVH) parameters, we evaluated the DVH parameters of the bowel bags of seven cases whose treatment planning data were available for analysis (Supplementary Table 2). A virtual conventional plan including the common iliac lymph node area as the CTV and a corresponding CS plan were generated. The bowel bag was contoured and then the DVH parameters after 20 Gy of pelvis irradiation plus 30 Gy of CS irradiation were calculated.

### Follow-up

Patients were followed-up every 1–3 months for the initial 2 years, and then every 3–6 months for the subsequent 3 years. The disease status and extent of late toxicities were assessed at each follow-up. Suspected recurrent cervical tumors were confirmed by biopsy wherever possible. Late toxicities were classified according to the Radiation Therapy Oncology Group (RTOG) and the European Organization for Research and Treatment of Cancer (EORTC) late toxicity criteria [10] with slight modification; diarrhea and colic were classified as toxicities of the small intestine, whereas bleeding was classified as toxicity of the large intestine/rectum. Local control (LC) was measured from the date of commencing therapy to the date of the first local recurrence or last follow-up. Progression-free survival (PFS) was measured from the date of commencing therapy to the date of the first pelvic disease progression (including local recurrence) or last follow-up. Overall survival (OS) was measured from the date of commencing therapy to the date of death from any cause or last follow-up. Survival analyses were conducted using SPSS software version 27.0 (SPSS, Chicago, IL, USA). The LC, PFS and OS rates were calculated using the Kaplan–Meier method.

## RESULTS

**Fig. 2.**
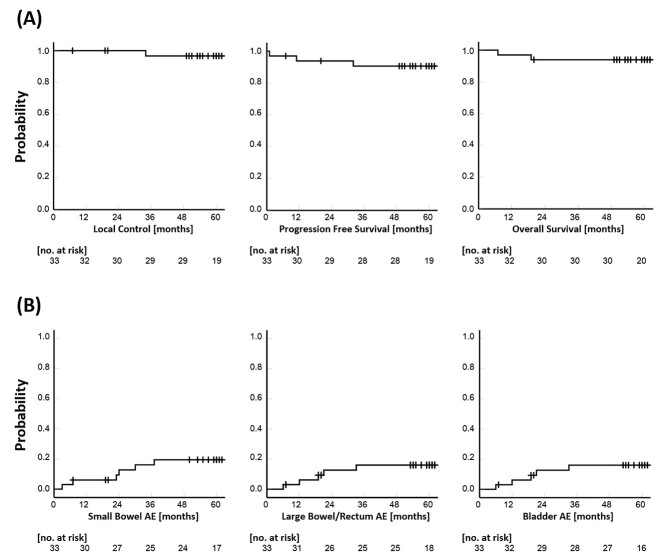
(A) Kaplan–Meier curves for LC, PFS and OS. (B) Kaplan–Meier curves for AEs of the small bowel, large bowel/rectum, and bladder.

Thirty-three patients with stage I–II cervical squamous cell cancer (≤4 cm) and without pelvic/para-aortic lymphadenopathy were treated with definitive radiotherapy with small-pelvis planning between February 2009 and September 2016. The patients’ characteristics are summarized in Table 1. With a median follow-up of 60.5 months (range, 7–105) for all patients, two patients developed distant recurrence and one developed a combination of local and distant recurrence (details are presented in Table 2). Of the 33 patients, two died because of cervical cancer, one from hepatocellular carcinoma, and one from non-cancerous disease. The 2- and 5-year LC, PFS and OS rates were 100% and 96.7%, 93.8% and 90.6%, and 93.9% and 93.9%, respectively (Fig. 2). No pelvic or para-aortic lymph node recurrence was observed.

**Table 1:** Summary of patient characteristics (*n* = 33)

Characteristic		
Age at diagnosis (years)		
median (range)	66	(43−88)
FIGO stage (2008)		
IB1	14	(42%)
IIA1	4	(12%)
IIB	15	(45%)
Lymph node		
Negative	33	(100%)
Chemotherapy		
No.	33	(100%)
Tumor size (cm)		
≤ 20	7	(21%)
20–40	26	(79%)
SCC (ng/ml)		
≤1.5	14	(42%)
>1.5	19	(58%)

Abbreviations: FIGO, Fédération Internationale de Gynécologie et d’Obstétrique; SCC, Squamous cell carcinoma antigen.

**Table 2:** Details of cases showing recurrence

Case	Age	FIGO stage	Tumor size	Recurrence site, (months)^†^	Post therapy	State (months)^†^
#1	66	IIB	30 mm	Lung (11)	Resection	NED (53)
#2	87	IIB	32 mm	Lung (32) Cervix (34)	Palliative RT to cervix and BSC	DOD (70)
#3	80	IB	10 mm	Liver (1)	BSC	DOD (19)

Abbreviations: BSC, best supportive care; NED, no evidence of disease; DOD, dead of disease.

^†^Time after start of initial RT.

Late complications (RTOG/EORTC) included five of grade 1 and two of grade 2 in the small intestine, five of grade 1 in the large intestine/rectum, and two of grade 1 and two of grade 2 in the bladder. No complication of grade 3 or higher was observed (Table 3). The 2- and 5-year late complication rates (G1 or higher) were 9.4% and 19.5%, respectively, for small intestine, 12.6% and 16% respectively for large intestine/rectum and 3.3% and 14.7%, respectively, for bladder (Fig. 2). The DVH parameters of the bowel bags of seven of the 33 cases were evaluated (Supplementary Table 2). The volumes of the bowel bags V15cc, V30cc, V40cc, and V45cc were reduced by using the small-pelvis irradiation field in all seven cases tested; after irradiation, the mean reduced volumes of the bowel bags were 159.2cc (V15), 89.8cc (V30), 73.7cc (V40), and 46.5cc (V45). Furthermore, pelvic insufficient fracture (IF), evaluated on CT images, was observed in eight of the 33 patients (24.2%), although symptomatic IF was only observed in four (12.1%) cases.

**Table 3:** Details of late complications^†^ (n = 33)

	RTOG/EORTC Grade					
	0	1	2	3	4	Total
Small Intestine^‡^	26	5	2	0	0	33
Large Intestine/Rectum^¶^	28	5	0	0	0	33
Bladder	29	2	2	0	0	33

^†^Some patients had complications in multiple organs.

^‡^Mild diarrhea *n* = 5, Moderate diarrhea and colic *n* = 2.

^¶^Slight rectal bleeding *n* = 5.

## DISCUSSION

This single-center retrospective study evaluated definitive radiotherapy consisting of small-pelvis irradiation and CT-based IGBT in stage I–II non-bulky cervical squamous cell carcinoma (≤ 4 cm). A landmark study of early-stage cervical cancer in Japan (JAROG0401/JROSG04-2: a prospective multi-institutional study of definitive radiotherapy using high-dose-rate intracavitary brachytherapy with a low cumulative dose schedule in non-bulky early-stage cervical cancer; Toita *et al.* [11]) reported radiotherapy consisting of whole pelvis irradiation of 20 Gy delivered in 10 fractions followed by pelvic irradiation with a midline block of 30 Gy delivered in 15 fractions, and intracavitary brachy therapy of 24 Gy delivered in four fractions. The 2- and 3-year pelvic disease PFS, disease free survival and OS rates were 96% and 96%, 90% and 90%, and 95% and 95%, respectively, which are comparable to those reported in the present study. These results suggest that a combination therapy of pelvic irradiation without treatment of the common iliac lymph nodes and IGBT can provide excellent LC without causing severe toxicity in patients with non-bulky (≤4 cm) cervical squamous cell carcinomas.

A previous dosimetric study demonstrated that small-pelvis irradiation significantly reduced the dose to the small bowel at any dose level (5 to 50 Gy) compared with conventional pelvic irradiation [4]. The important goal of small-pelvis irradiation is reduction of adverse events (AEs) involving the small bowel; our observations showed that the 2- and 5-year small bowel AE rates in grades 1 and 2 were 12.8% and 19.5%, respectively. Notably, for grade 3 or higher, no AEs were observed. Late complications (Grade 2) of 1.7% (1 of 60 patients) in the small intestine, 3.3% (two of 60 patients) in the large intestine/rectum, and 1.7% (one of 60 patients) in the bladder were also reported in the JAROG0401/JROSG04-2 study, but no complications of grade 3 or higher were reported. These low incidences can be attributed to the low cumulative doses delivered to the central pelvis. In our study, late complications (Grade 2) were 6.6% (two of 33 patients) in the small intestine, 0% (none of 33 patients) in the large intestine/rectum and 6.6% (two of 33 patients) in the bladder, indicating that the low incidence of complications can be reproduced even after 5 years of observation.

Isohashi *et al.* reported useful DVH parameters that predict G3 chronic GI complications after postoperative radiotherapy for cervical cancer [12]. Receiver Operating Characteristic (ROC) analysis revealed that the optimal thresholds in the bowel bag were 940 cc in V30 (*P* = 0.06, odds ratio 3.24), 850 cc in V40 (*P* <0.01, odds ratio 7.10) and 800 cc in V45 (*P* = 0.01, odds ratio 4.59). Among our seven cases, V30 and V40 in case D and V30 in case G exceeded the thresholds in the conventional plans, but not in the small plans, which may reduce the incidence of late complications. Furthermore, the incidence of small bowel AEs is known to increase with prolonged observation over more than 10 years (the grade 3 or higher AEs of small bowel at 5 years, 10 years and 20 years are 2.6%, 3.3% and 8.3%, respectively) [13], further long-term observation is warranted to confirm the clinical benefit of small-pelvis irradiation.

Patient selection is important in the use of small-pelvis plans that exclude the common iliac lymph node region, as it is important not to undermine the curability of the disease. Squamous cell carcinoma of the cervix, which constitutes approximately 80% of all cervical cancers, commonly metastasizes to the lymph nodes, moving in an anterograde direction along vessels from the paracervical lymph nodes to the common iliac lymph nodes. Sakuragi *et al.* reported that in 208 patients with stage IB to IIB surgically-treated cervical cancer, pelvic lymph node metastasis was found in 25.5% of cases, most frequently involving the obturator lymph node (18.8%) and common iliac lymph node (9.1%). Furthermore, the presence of pelvic lymph node metastasis was correlated with the local extent of the tumor [14], supporting the concept of anterograde metastasis. The frequency of common iliac lymph node metastasis is less in lower-stage disease, in which the local extent of the tumor is also lower. Siu *et al.* reported that in 174 patients with stage IA to IIA disease, the rates of pelvic lymph node metastasis and common iliac lymph node metastasis were 20.1% and 4.6%, respectively [15], while Li *et al.* reported that in 665 patients with stage IA to IIA disease, the rates of pelvic lymph node metastasis and common iliac lymph node metastasis were 25.3% and 4.6%, respectively [16]. In another study, the rates of pelvic node and common iliac lymph node metastasis were 21% and 4.2%, respectively, but only 16.7% and 2.4% for a tumor size of ≤4 cm [5]. This indicates our reasoning for omitting the common iliac lymph node area from the pelvic irradiation field. In the EMBRACE II study, nodal targeting is also defined according to the risk of nodal spread, with the proposed omission of the common iliac node region in early disease cases; however, the clinical outcomes of this study are not yet reported [17]. In our 33 patients, three cases had recurrence, but neither the pelvic nor para-aortic lymph nodes were found to have recurrence, suggesting the feasibility of our treatment strategy.

On the other hand, small-pelvis irradiation may reduce pelvic IF by reducing the radiation dose to the pelvic bones [4]. In our study, pelvic IF evaluated with CT images was observed in eight of the 33 patients (24.2%), although symptomatic IF was observed in only four (12.1%) cases. Tokumaru *et al.* reported that pelvic IF and symptomatic pelvic IF occurred in 36.9% and 16.1%, respectively, of patients who received definitive radiotherapy for early-stage uterine cervical cancer [18]. The effect of small-pelvis irradiation on pelvic IF will need to be clarified further in a larger cohort study.

Discussion of the three cases of recurrence is warranted. Distant metastasis was observed in three patients, two with lung metastasis and one with liver metastasis (Table 2). In one patient, lung metastasis was resected and pathologically proven to be metastatic; the patient is still alive with no evidence of recurrence at 53 months after resection. Local recurrence was observed in only one case, which was from the center of the cervix (34 months) and was associated with lung metastasis (32 months). The total dose of HR-CTV D90% from four brachytherapy sessions in this case was 27.08 Gy. The cervical tumor responded to 39 Gy/13 fractions of local palliative radiotherapy, and the patient survived until 70 months. A few squamous cell carcinomas develop distant metastasis without lymph node metastasis. Therefore, as a proportion of such cases are salvageable with local treatment, frequent follow-up including chest CT is needed. In the near future, genetic testing (e.g. liquid biopsy) [19] or immune-scoring of tumor specimens [20] may identify tumors that metastasize in the early stage.

There are several limitations to this study. First, as the study included a relatively small number of patients, a larger study is needed to confirm our observations of low rates of AEs and treatment efficacy. Second, there are biases that cannot be removed because of the retrospective nature of the study. Our results should be validated in a prospective study enrolling a larger cohort.

## CONCLUSION

Our results suggest that combination therapy of small-pelvis irradiation excluding the common iliac lymph node plus IGBT provides reasonable clinical outcomes and can be considered a treatment option in non-bulky (≤4 cm) cervical cancer.

## CONFLICT OF INTEREST

The authors confirm they have no conflicts of interest to declare.

## FUNDING

This work was supported by grants-in-aid from the Ministry of Education, Culture, Sports, Science, and Technology of Japan [KAKENHI; grant number 26 461 879 to T.O.].

## PRESENTATION AT A CONFERENCE

A part of the work described in this study has been presented at the 32nd annual meeting of JASRO, 2019.

## Supplementary Material

revised_supplementary_table_1_rrac001

revised_supplementary_table_2_rrac001
