# Supplementary material for: A retrospective study of small-pelvis radiotherapy plus image-guided brachytherapy in stage I–II non-bulky cervical squamous cell carcinoma
Source: J Radiat Res. 2022 Feb 12;63(2):290–5. doi: 10.1093/jrr/rrac001 (PMC8944301; doi:10.1093/jrr/rrac001)
Supplement: revised_supplementary_table_1_rrac001 [file revised_supplementary_table_1_rrac001.pptx]

## Slide 1
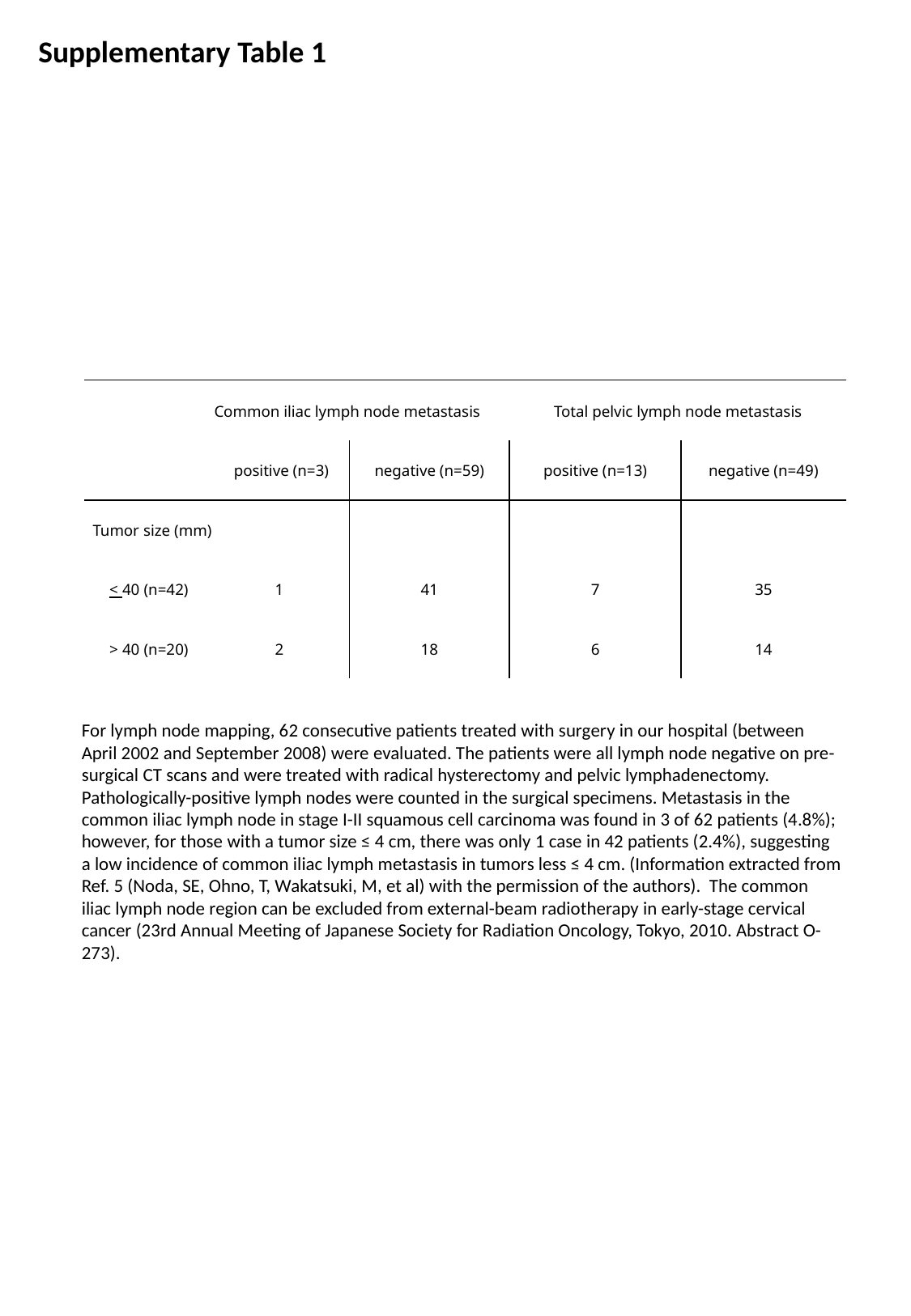

Supplementary Table 1
| | Common iliac lymph node metastasis | | | Total pelvic lymph node metastasis | |
| --- | --- | --- | --- | --- | --- |
| | positive (n=3) | | negative (n=59) | positive (n=13) | negative (n=49) |
| Tumor size (mm) | | | | | |
| < 40 (n=42) | 1 | | 41 | 7 | 35 |
| > 40 (n=20) | 2 | | 18 | 6 | 14 |
For lymph node mapping, 62 consecutive patients treated with surgery in our hospital (between April 2002 and September 2008) were evaluated. The patients were all lymph node negative on pre-surgical CT scans and were treated with radical hysterectomy and pelvic lymphadenectomy. Pathologically-positive lymph nodes were counted in the surgical specimens. Metastasis in the common iliac lymph node in stage I-II squamous cell carcinoma was found in 3 of 62 patients (4.8%); however, for those with a tumor size ≤ 4 cm, there was only 1 case in 42 patients (2.4%), suggesting a low incidence of common iliac lymph metastasis in tumors less ≤ 4 cm. (Information extracted from Ref. 5 (Noda, SE, Ohno, T, Wakatsuki, M, et al) with the permission of the authors). The common iliac lymph node region can be excluded from external-beam radiotherapy in early-stage cervical cancer (23rd Annual Meeting of Japanese Society for Radiation Oncology, Tokyo, 2010. Abstract O-273).
