# Supplementary material for: A retrospective study of small-pelvis radiotherapy plus image-guided brachytherapy in stage I–II non-bulky cervical squamous cell carcinoma
Source: J Radiat Res. 2022 Feb 12;63(2):290–5. doi: 10.1093/jrr/rrac001 (PMC8944301; doi:10.1093/jrr/rrac001)
Supplement: revised_supplementary_table_2_rrac001 [file revised_supplementary_table_2_rrac001.pptx]

## Slide 1
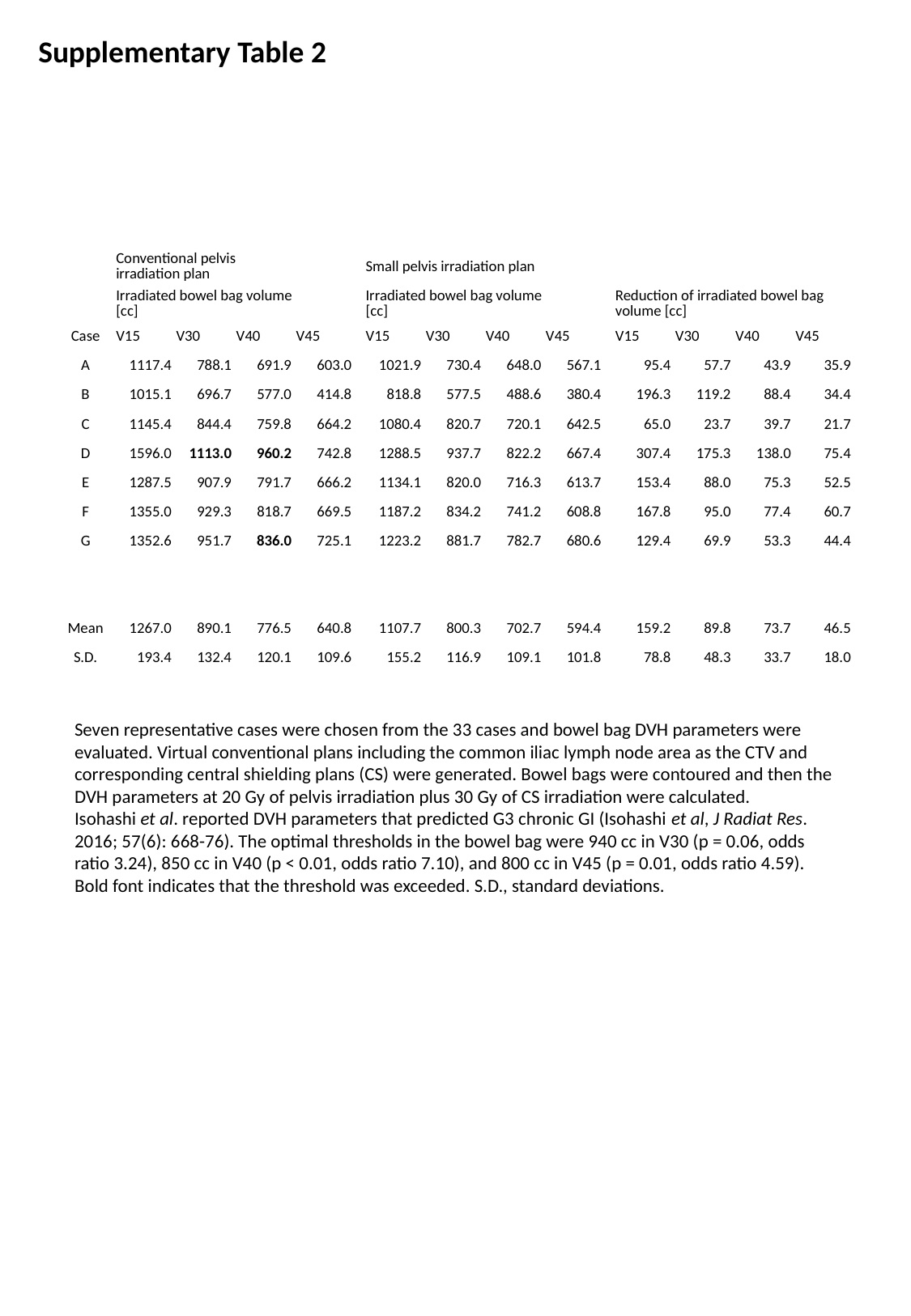

Supplementary Table 2
| | Conventional pelvis irradiation plan | | | | | Small pelvis irradiation plan | | | | | | | | |
| --- | --- | --- | --- | --- | --- | --- | --- | --- | --- | --- | --- | --- | --- | --- |
| | Irradiated bowel bag volume [cc] | | | | | Irradiated bowel bag volume [cc] | | | | | Reduction of irradiated bowel bag volume [cc] | | | |
| Case | V15 | V30 | V40 | V45 | | V15 | V30 | V40 | V45 | | V15 | V30 | V40 | V45 |
| A | 1117.4 | 788.1 | 691.9 | 603.0 | | 1021.9 | 730.4 | 648.0 | 567.1 | | 95.4 | 57.7 | 43.9 | 35.9 |
| B | 1015.1 | 696.7 | 577.0 | 414.8 | | 818.8 | 577.5 | 488.6 | 380.4 | | 196.3 | 119.2 | 88.4 | 34.4 |
| C | 1145.4 | 844.4 | 759.8 | 664.2 | | 1080.4 | 820.7 | 720.1 | 642.5 | | 65.0 | 23.7 | 39.7 | 21.7 |
| D | 1596.0 | 1113.0 | 960.2 | 742.8 | | 1288.5 | 937.7 | 822.2 | 667.4 | | 307.4 | 175.3 | 138.0 | 75.4 |
| E | 1287.5 | 907.9 | 791.7 | 666.2 | | 1134.1 | 820.0 | 716.3 | 613.7 | | 153.4 | 88.0 | 75.3 | 52.5 |
| F | 1355.0 | 929.3 | 818.7 | 669.5 | | 1187.2 | 834.2 | 741.2 | 608.8 | | 167.8 | 95.0 | 77.4 | 60.7 |
| G | 1352.6 | 951.7 | 836.0 | 725.1 | | 1223.2 | 881.7 | 782.7 | 680.6 | | 129.4 | 69.9 | 53.3 | 44.4 |
| | | | | | | | | | | | | | | |
| | | | | | | | | | | | | | | |
| Mean | 1267.0 | 890.1 | 776.5 | 640.8 | | 1107.7 | 800.3 | 702.7 | 594.4 | | 159.2 | 89.8 | 73.7 | 46.5 |
| S.D. | 193.4 | 132.4 | 120.1 | 109.6 | | 155.2 | 116.9 | 109.1 | 101.8 | | 78.8 | 48.3 | 33.7 | 18.0 |
Seven representative cases were chosen from the 33 cases and bowel bag DVH parameters were evaluated. Virtual conventional plans including the common iliac lymph node area as the CTV and corresponding central shielding plans (CS) were generated. Bowel bags were contoured and then the DVH parameters at 20 Gy of pelvis irradiation plus 30 Gy of CS irradiation were calculated.
Isohashi et al. reported DVH parameters that predicted G3 chronic GI (Isohashi et al, J Radiat Res. 2016; 57(6): 668-76). The optimal thresholds in the bowel bag were 940 cc in V30 (p = 0.06, odds ratio 3.24), 850 cc in V40 (p < 0.01, odds ratio 7.10), and 800 cc in V45 (p = 0.01, odds ratio 4.59). Bold font indicates that the threshold was exceeded. S.D., standard deviations.
